# Dispositional mindfulness, anticipation and abstinence symptoms related to hypnotic dependence among insomniac women who seek treatment: A cross-sectional study

**DOI:** 10.1371/journal.pone.0194035

**Published:** 2018-03-16

**Authors:** Víviam Vargas Barros, Emérita Sátiro Opaleye, Marcelo Demarzo, Sarah Bowen, Daniela Fernández Curado, Helena Hachul, Ana Regina Noto

**Affiliations:** 1 Nepsis – Research Center on Health and Substance Use – MBRP Brasil – Brazilian Center for Research and Training in Mindfulness-Based Relapse Prevention, São Paulo, Brazil; 2 Department of Psychobiology, Universidade Federal de São Paulo, São Paulo, São Paulo, Brazil; 3 Mente Aberta – Brazilian Center for Mindfulness and Health Promotion - Department of Preventive Medicine, Universidade Federal de São Paulo, São Paulo, São Paulo, Brazil; 4 Hospital Israelita Albert Einstein, São Paulo, Brazil; 5 Psychology Department, Pacific University, School of Health Professions. Hillsboro, Oregon, United States of America; 6 Department of Gynecology & Obstetrics, Casa de Saúde Santa Marcelina, São Paulo, Brazil; Charité - Universitätsmedizin Berlin, GERMANY

## Abstract

**Introduction:**

Dispositional mindfulness can be described as the mental ability to pay attention to the present moment, non-judgmentally. There is evidence of inverse relation between dispositional mindfulness and insomnia and substance use, but as of yet, no studies evaluating the specific association between dispositional mindfulness and the components of hypnotic use disorder.

**Objective:**

To evaluate the association between dispositional mindfulness and the components of dependence among female chronic hypnotic users.

**Design and method:**

Seventy-six women, chronic users of hypnotics, who resorted to *Mindfulness-Based Relapse Prevention* for the cessation of hypnotic use were included in the study. The *Five Facet Mindfulness Questionnaire (FFMQ)* evaluated the levels and facets of mindfulness, and the subscales of the *Benzodiazepine Dependence Questionnaire (BENDEP)* assessed dependence on hypnotics. We also evaluated sociodemographic variables and symptoms of insomnia and anxiety. The associations between the FFMQ facets and the BENDEP subscales were evaluated with binomial logistic regression, adjusted for income, schooling, anxiety, and insomnia.

**Results:**

We observed associations between facets of the FFMQ and specific aspects of hypnotic dependence. The facet “observing” was inversely associated with the “concern about lack of availability of the hypnotic” [aOR = 0.87 95% CI (0.79–0.97)], and the facet “non-reacting to inner experience” with “noncompliance with the prescription recommendations” [aOR = 0.86 95% CI (0.75–0.99)]. The total score of the FFMQ was inversely associated to those two dependence subscales [aOR = 0.94 95% CI (0.89–0.99)]. “Observing” and “non-reactivity to inner experience” were also inversely associated with the “impairments related to the withdrawal symptoms” [aOR = 0.84 95% CI (0.73–0.97)] and [aOR = 0.78 95% CI (0.63–0.96)], respectively. The FFMQ was not associated with *“*awareness of problematic hypnotic use”.

**Conclusion:**

Dispositional mindfulness, specifically the facets “observing” and “non-reactivity to inner experience, were inversely associated with the components of hypnotic dependence related to the anticipation of having the substance, its expected effect, and the impairments caused by the abstinence. We discuss the implications of those results for the clinical practice and future investigations.

## Introduction

Hypnotics, mainly benzodiazepines (BZD), are the most highly prescribed class of psychopharmacological medication worldwide [1]. They are the leading prescription in Brazil, with an especially high number of prescriptions among women [2]. The use of BZD for the treatment of primary disorders such as sleep disturbances and anxiety for more than four weeks, even in therapeutic doses, can cause cognitive problems, psychomotor and memory impairment, in addition to tolerance, dependence and withdrawal syndrome [3]. Following advancement in research on the deleterious effects of benzodiazepines, prescriptions for hypnotics other than benzodiazepines, such as zolpidem, zopiclone and ezopiclone (known as z drugs), began to rise. As these are newer drugs, and subject of fewer studies, their long-term deleterious effects are unclear. However, since their mechanism of action is quite similar to that of benzodiazepines (GABA-A agonists, although not specific of the benzodiazepine site) [4], further studies are required to evaluate the risks associated to consumption. There are already reports in the literature suggesting acute cognitive side effects of z drugs [5] and that continuous exposure to z drugs, as well as to benzodiazepines, significantly increase the odds of mortality, even when adjusted for other mortality factors [6, 7]. The term “hypnotics” will be used in this study as a reference to both classes of medication, indiscriminately.

Studies suggest a belief among physicians that hypnotics do not cause serious damage, and lead to dependence only when used at high doses. There is a perceived lack of time and of pharmacological alternatives to treat insomnia patients; therefore, many prescribers believe hypnotics are one of the few treatment options [8]. Consequently, although most use is in accordance with a prescription, medical supervision may not include knowledge of or information given regarding the risks of prolonged use. This may lead to chronic consumption, based on patient belief that it is safe [9]. Moreover, as the association among insomnia, anxiety and daytime hyperarousal is frequent [10], psychological and cognitive factors also foster chronic use. For instance, anticipation of negative effects due to a bad night of sleep on daytime functioning leads to higher levels of anxiety that may in turn lead to preoccupation about the availability of the hypnotic, and even to the use of a higher dose [11]. Similarly, intolerance to the withdrawal symptoms when the medication is discontinued increases risk of chronic use [12].

One of the few instruments that considers these important components of hypnotic dependence is the Benzodiazepine Dependence Questionnaire (BENDEP-SRQ), which defines dependence as having four components: (1) Problematic use, (2) Preoccupation with the availability of the medicine, (3) Lack of compliance with the prescription, and (4) Impairments caused by withdrawal. The subscales “Preoccupation” and “Lack of compliance” both assess anticipatory anxiety, or thoughts of potential consequences of running out of, not using, or not having a high enough dose of the medication. In populations with higher levels of anxiety, these anticipatory thoughts may foster: 1) excessive and uncontrollable concern in the form of ruminating thoughts that perpetuate and increase anxiety, leading to a “descending spiral”; 2) compulsive or automatic actions to alleviate anxiety, often accompanied by self-judgment which may also perpetuate the descending spiral [13, 14]. Individuals often believe these cognitions and behaviors are productive, thus sustaining cycles of use [15].

Awareness of the role that cognitive patterns, such as anticipatory anxiety, play in the onset and maintenance of insomnia, and a kinder attitude towards discomfort caused by these thoughts, or by withdrawal symptoms from reduction/removal of the hypnotic, might contest the avoidance-based coping patterns [16] fostering alternative and effective coping responses [17]. Mindfulness may be a valuable skill for individuals who present those symptoms. Mindfulness includes metacognitive ability described as “paying attention in a particular way; on purpose, in the present moment, and non-judgmentally" [18]. Through mindfulness training, this innate ability can be strengthened [19].

The practice of mindfulness was initially incorporated into the Western medicine as a complementary approach to help patients better handle stress, chronic pain, and other clinical conditions [18]. Literature on mindfulness for other conditions as insomnia, anxiety, and substance use is quickly growing [20–22]. Higher levels of dispositional mindfulness (innate ability or psychological trait) fosters a better relation with the cognitive or physical discomfort, resulting from those disorders [23]. In populations with anxiety and substance dependence, mindfulness increases awareness of triggers of automatic reactions, such as anticipatory rumination [24] or substance use [25], and trains individuals to disengage from unhelpful thoughts, thus allowing them have more skillful choices to cope with challenges [26].

Several questionnaires aim to evaluate levels of dispositional mindfulness. Recent studies have used measures to evaluate components of the mindfulness, encompassing not only attention, but also non-judgment, non-reactivity, and openness to the experience [27]. Among these measures, the *Five Facet Mindfulness Questionnaire* (FFMQ), evaluating five components of mindfulness [28], is one of the most widely used. It has been validated in several countries [29–33], including Brazil [34].

The objective of the current cross-sectional study was to evaluate the association between dispositional mindfulness and insomnia in treatment seeking female hypnotics users. Specifically, the study assessed facets of mindfulness and their relation to components of hypnotic dependence assessing anticipation of availability or effects of hypnotics. In line with previous studies evincing a negative association between mindfulness facets and psychiatric symptoms [35], our hypotheses were that mindfulness, specifically the “observing” and “non-reactivity” facets, would be inversely associated with the subscales of hypnotics dependence assessing the anticipatory component, and the impairments caused by withdrawal.

## Materials and method

### Participants

The sample of the present cross-sectional study used data from a randomized clinical trial evaluating the efficacy of a Mindfulness-Based Relapse Prevention (MBRP) on reduction of hypnotic use among female chronic users (ClinicalTrials.gov Identifier: NCT02127411). The sample was composed of women residing in Sao Paulo, Brazil, recruited via print and digital media, radio, and television. Recruiting and selection used the following inclusion and exclusion criteria:

### Inclusion criteria

- Adult women, over 18 years of age, able to read and write in Portuguese
- Use of hypnotic medication for sleep induction for at least three months (90 days), at least four times a week, as assessed by a questionnaire assessing the pattern of hypnotics use.

### Exclusion criteria

- Presence of neurologic conditions, cancer, anxiety refractory to other treatments, base psychiatric disorders, secondary insomnia or other severe clinical conditions for which cessation of the hypnotic would present risk of worsening
- Dependence on or abuse use of alcohol or other drugs, except tobacco
- Undergoing acute treatment for psychological or psychiatric disorders
- Current participation in a program or protocol for hypnotic cessation
- Engagement in yoga, meditation, or other contemplative practices in the six months prior to data collection

Interested individuals (234) underwent a brief phone screening. Of 234 respondents, 118 women did were deemed ineligibility due to: chronic use of hypnotics (n = 55); engagement in contemplative practices in past six months (n = 29); participation in psychiatric treatment for acute depression, panic disorder, bipolar disorder, substance dependence or generalized anxiety (n = 18); lack of time or availability to participate in research activities (n = 12); or declined participation but did not provide a reason (4).

**Fig 1:**
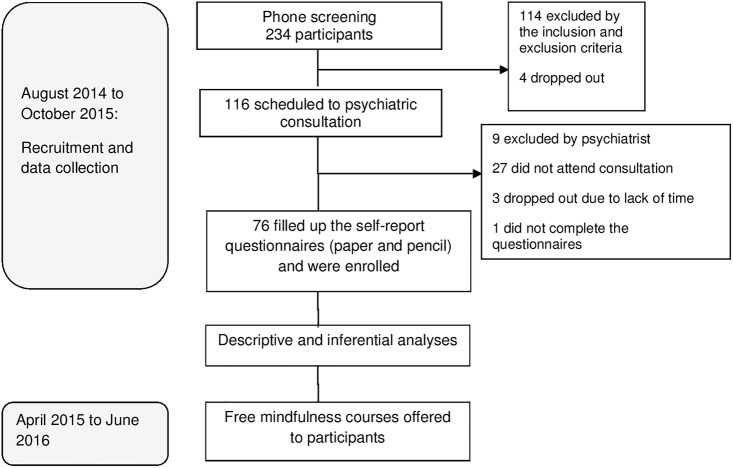
Flowchart of the study procedures.

In the second phase of the screening, the remaining 116 potentially eligible women were referred to a psychiatric consultation. Exclusions in this phase were predominantly due to a clinical condition that could destabilize with the withdrawal of the hypnotic, resulting in a final baseline sample of 76 participants. See Fig 1 for recruitment flow diagram.

### Procedures

After screening, eligible participants were invited to the Universidade Federal de São Paulo (UNIFESP) for data collection. The current cross-sectional study used paper-and-pencil self-report data collected at baseline in the presence of one research team member. The questionnaire battery took approximately 50 minutes to complete. Baseline data collection occurred between August 2014 and October 2015.

We conducted this study in accordance with the Declaration of Helsinki, and the Committee of Ethics in Research (CER) of the Universidade Federal de São Paulo approved all the procedures (CAAE: 53377116.7.0000.5505). Participants were informed on the risk of discomfort while filling out some of the questionnaires, and compensation in the form of a free 16-hour mindfulness course. All the participants signed an informed consent form.

#### Instruments

After being screened into the study, participants answered a sociodemographic questionnaire assessing age, education, family income, marital status, who they live with, profession, menopause status, and medical supervision of hypnotics use. Additionally, they answered the following structured questionnaires:

#### Dependence on hypnotics

Dependence on hypnotics was evaluated by the Benzodiazepine Dependence Self-Report Questionnaire Portuguese Version (BENDEP-SRQ-PV) Questionnaire, short Portuguese version, translated and adapted to Brazilian Portuguese, under validation by the authors of the present study. The scale evaluates the level of perception users have of their dependence, using four subscales (terms in parentheses will henceforth be used for abbreviation): *Problematic use* (Problematic use), or level of awareness of the problematic use (α = 0.54); *Preoccupation about the availability* (Preoccupation), or level of concern/obsession about the availability of the hypnotic (α = 0.61); *Lack of compliance with the prescription* (Lack of compliance), or level of lack of compliance with the therapeutic regimen (α = 0.63); and *impairments induced by the withdrawal of the medication* (Withdrawal) or level of certainty in relation to the damage perceived associated with withdrawal symptoms (α = 0.68) [36]. All the items are evaluated on a five-point Likert scale.

#### Dispositional mindfulness

The Five Facet Mindfulness Questionnaire (FFMQ) [28] has been validated in Brazil [34] and has shown good internal consistency (α = 0.81, in this sample α = 0.85). It consists of 39 items on a five-point Likert scale that ranges from “Never or rarely true” to “Very often or Always true”. The Brazilian version comprises seven factors that evaluate five domains of Mindfulness. The factors and Cronbach’s α coefficients of this sample were as follows: a) Observing (8 items): perception of internal and external experiences such as sensations, cognitions, emotions, sights, sounds and smells (α = 0.79); b) Describing (8 items): capacity of naming inner experiences, with positive and negative formulation in the Brazilian version (positive α = 0.78; negative α = 0.83; c) Non-reactivity to inner experiences (7 items): perception of thoughts and feelings without engaging in or being carried away by them (α = 0.73); d) Acting with awareness (8 items): capacity of being focused on the activities of the moment as opposed to the “automatic pilot”, that is, engaging mechanically in activities while having attention focused elsewhere. In the Brazilian validation, items in this factor were further divided into two subfactors, one with items related to automatic pilot (α = 0.80) and the other with items related to distraction (α = 0.73); e) Non-judging of inner experience (8 items): related to not evaluating and judging feelings and thoughts (α = 0.84).

#### Insomnia

Insomnia severity was assessed by the Insomnia Severity Index (ISI) [37], validated in Brazil with good psychometric indicators (α = 0.86) [38]. The questionnaire consists of seven items that evaluate severity of sleep onset, sleep maintenance and early morning awakening problems, sleep dissatisfaction, interference of sleep difficulties with daytime functioning, noticeability of sleep problems by others, and distress caused by sleep difficulties. The score for each item ranges from 0 to 4, for a total possible range of 0 to 28 points, with a higher score indicating greater insomnia severity. Cronbach’s α of the scale in the current sample was identical to that of the original validation study in Brazil (α = 0.86).

#### Anxiety

The State and Trait Anxiety Index (STAI) [39] has been validated in Brazil (α = 0.87) [40]. The current study used only the trait anxiety subscale, which is less susceptible to environmental alterations. This subscale is composed by 20 items on a four-point Likert scale (“almost never”, “sometimes”, “often”, “almost always”), in which the participant is asked, “How do you usually feel?”. Cronbach’s α in this sample was excellent (0.90).

#### Statistical analyses

For the descriptive analyses, we presented medians with interquartile intervals (IQR), the amplitude of the distribution for the continuous data, and description of the frequency for the categorical data (see Table 1).

**Table 1:** Sociodemographic and hypnotics use characteristics (*N* = 76).

		Median	IQR	Min-Max
**Age group**		50	15	25–85
**Time using hypnotics (months)**		30	51	3–264
**Marital status**		**n**		**%**
Single		18		23.7
Married		34		44.7
Separated/Divorced/Widowed		23		30.3
Missing data		1		1.3
**Lived with their children**		**35**		**46.7**
**Schooling**		**n**		**%**
Incomplete to complete junior high		10		13.2
Incomplete to complete high school		19		25
Incomplete college to post-graduation		46		60.5
Missing data		1		1.3
**Family Income per month**^2^		**n**		**%**
U$ 154–618		11		14.5
U$ 618–1545		29		38.2
U$ 1545–3090		17		22.4
More than U$ 3090		13		17.1
Missing data		6		7.9
**Main income source of the family**		**n**		**%**
No		37		48.7
Yes		36		47.4
Missing data		3		3.9
**Menopause**		**n**		**%**
No		20		26.3
Yes		54		71.0
Missing data		2		2.6
**Medical supervision for the use of hypnotics**		**n**		**%**
No supervision, use at their own risk		10		13.2
Yes		64		84.2
Missing data		2		2.6
**The physician evaluates sleep or the deleterious effects of hypnotics**		**n**		**%**
No, he/she only renews the prescription.		32		42.1
Yes		40		52.6
Missing data		4		5.3
**Number of hypnotics used**		**n**		**%**
1		67		88.2
2 or more		9		11.8
**Ever tried to reduce or stop use**		**n**		**%**
No		13		17.1
Yes		63		82.9
**Hypnotic used**^1^	**Daily dose Min-Max**	**n**		**%**
Diazepam	10-20mg	2		2.7
Lorazepam	1-4mg	5		6.7
Alprazolam	0.15-4mg	15		20
Clonazepam	0.1-10mg	29		38.7
Zolpidem	0.25-40mg	31		41.3
Other^3^	1-15mg	4		5.3

^1^Percentage does not reach 100% because the variable yields more than a single response.

^2^A monthly minimum wage in Brazil corresponded to approximately US$ 309,00 by the time of the data collection.

^3^The other hypnotics used were Bromazepam, Cloxazolam, Chlordiazepoxide and Zopiclone

We initially categorized the subscales of the BENDEP-SRQ-PV into binary variables to increase the statistical power of the analyses. We carried out Generalized Linear Models (GzLM) for the binary dependent variable (binomial regression) with binomial distribution for all the BENDEP-SRQ-PV subscales. To choose the distribution, we used the lowest value found for the Akaike Index Criteria (AIC) among the Poisson, binomial and negative binomial models.

To assess primary study outcomes, we performed simple regressions containing outcome variables (factors of the hypnotic dependence scale) and other variables that would be potentially associated (mindfulness, anxiety, insomnia, schooling, income, age, type of hypnotic, time of use and use under medical supervision). We then tested several binomial regression models to evaluate the association between each of the BENDEP-SRQ-PV factors and associated variables as established in the simple regressions. The models were adjusted for variables that might interfere in the use of hypnotics or in the comprehension of the questionnaires (i.e., continuous variables: insomnia severity, age and anxiety; categorical variables: schooling and income). Menopause was not included in the adjustment because it presented multicollinearity with age. All regression models included only complete data. The minimum *n* included in models was 68 participants, or 89.5% of the sample. The subscale “Whithdrawal” of the BENDEP-SRQ-PV only includes women who had tried to reduce the medication previously; therefore, models including this variable had at least 58 of the 63 women that answered this subscale, totaling 93.1% of this sample.

We performed analyses of normality of residuals based on qq plot graphs to evaluate the quality of the final model. All the analyses were performed on the software STATA version 12, with the level of significance set at .05. According to the residual analysis by the qq plot graphs of tested models, the residuals followed normal distribution, indicating the good model quality.

## Results

### Characteristics of the sample

Descriptive analyses are included in Table 1. All participants (*N* = 76) were female, with median age of 50 years (IQR = 15), and a majority had at least started college (60.5%).

Over a third reported a monthly income varying from (in U.S. dollars) $618.00 to $1,545.00 (38.2%) or from $1,545.00 to $3,090.00 (22.4%), and the income was shared among up to three people in 85.3% of the cases. Only 20.5% of those women provided the household with the total monthly income, that is, they were the main income source and, in 48.7% of the other cases, some other person provided the household with the major income.

Concerning hypnotics, the median of age of onset of use was 48 years (IQR = 19), with continued use for 30 months (IQR = 51), and a minimum time of three months and maximum of 264 months. Regarding sites under which they had hypnotic use supervision, 57.3% received service at a private physician’s office, 20% at a Basic Health Unit (UBS), 8.0% in hospitals and 13.3% had no supervision. Of those with no supervision, 50% procured the medication through an acquaintance or a physician in the family, 41.7% through friends or family members, and 8.3% purchased it on the internet. Of those who were supervised, in 44.4% the physician did not evaluate the patient’s sleep or the possible deleterious effects of the long-term use of the hypnotic.

Tables 2 and 3 describe the data outcome and predictive variables, respectively.

**Table 2:** Descriptive data of the subscales of hypnotic dependence—Outcome variables.

**Bendep-SRQ-PV subscales**	n	%
**Problematic use**		
Very low to moderate	11	14.7
High to very high	63	82.7
Missing data	2	2.7
**Preoccupation**	**n**	**%**
Very low to moderate	37	48.7
High to very high	36	47.4
Missing data	3	3.9
**Lack of compliance**	**n**	**%**
Low	31	40.8
High to very high	43	56.6
Missing data	2	2.6
**Withdrawal**^a^	**n**	**%**
Low to very low	26	41.3
Moderate to high	37	58.7

^a^ The n of this variable included only women who had already tried to reduce or stop the use of hypnotics (n = 63); therefore, models including this variable had at least 58 of the 63 women that answered this subscale, totaling 93.1% of this sample.

**Table 3:** Descriptive data of the mindfulness facets, insomnia, and anxiety—Predictor variables.

	Median	IQR	Min—Max
**Insomnia severity**	18	7	5–28
**Total mindfulness**	94.5	25	53–126
Non-judging of inner experience	26	11	8–39
Describing (items with positive formulation)	15	7	6–23
Describing (items with negative formulation)	12	5	3–15
Non-reactivity to inner experience	17	6.5	7–30
Acting with awareness (automatic pilot)	17	4.5	7–21
Acting with awareness (distraction)	10	6	3–15
Observing	24.5	9	10–39
**Trait Anxiety**	51	14	24–72

We performed binomial regression analyses (GzLM) with hypnotic type (BZD or Z drug) as a predictor, and subscales of hypnotic dependence as outcomes, controlling for insomnia severity, age, schooling, income, and trait anxiety. No significant differences emerged between the two types of medication and risk of dependence subscales (Table 4).

**Table 4:** Association between dispositional mindfulness, type of hypnotic and hypnotic dependence.

**Problematic Use**	**n**	**Crude OR (95% CI)**	**p**	**n**	Adjusted* **OR (95%CI)**	**p**
Total FFMQ	68	0.98 (0.94–1.03)	0.458	60	1.00 (0.94–1.07)	0.848
Non-judging of inner experience	69	0.97 (0.89–1.07)	0.605	60	1.02 (0.87–1.20)	0.775
Describing (items with positive formulation)	70	0.99 (0.85–1.16)	0.947	61	1.02 (0.86–1.21)	0.817
Describing (items with negative formulation)	70	0.85 (0.66–1.08)	0.182	61	0.90 (0.66–1.23)	0.505
Non-reactivity to inner experience	70	0.98 (0.86–1.11)	0.697	61	0.96 (0.81–1.28)	0.601
Acting with awareness (automatic pilot)	70	0.99 (0.80–1.22)	0.914	61	1.07 (0.80–1.44)	0.636
Acting with awareness (distraction)	69	1.01 (0.83–1.23)	0.895	61	1.14 (0.89–1.45)	0.284
Observing	70	0.99 (0.89–1.09)	0.831	61	0.98 (0.86–1.11)	0.724
Type of hypnotic	73	1.26 (0.33–4.77)	0.730	62	3.12 (0.51–19.23)	0.219
**Preoccupation**	**n**	**Crude OR (95% CI)**	**p**	**n**	Adjusted* **OR (95%CI)**	**p**
Total FFMQ	68	0.94 (0.90–0.97)	0.001	60	0.94 (0.89–0.99)	0.020
Non-judging of inner experience	69	0.90 (0.83–0.97)	<0.01	60	0.90 (0.80–1.01)	0.085
Describing (items with positive formulation)	70	0.87 (0.77–0.97)	<0.05	61	0.90 (0.79–1.02)	0.098
Describing (items with negative formulation)	70	0.87 (0.75–1.02)	0.082	61	0.81 (0.65–1.00)	0.056
Non-reactivity to inner experience	70	0.97 (0.88–1.06)	0.583	61	0.99 (0.87–1.12)	0.852
Acting with awareness (automatic pilot)	70	0.86 (0.74–1.01)	0.067	61	0.95 (0.77–1.17)	0.630
Acting with awareness (distraction)	69	0.86 (0.74–0.99)	<0.05	61	0.92 (0.76–1.10)	0.340
Observing	70	0.89 (0.82–0.97)	<0.01	61	0.87 (0.79–0.97)	0.011
Type of hypnotic	73	1.31 (0.52–3.35)	0.567	62	1.82 (0.57–5.84)	0.314
**Withdrawal**	**n**	**Crude OR (95% CI)**	**p**	**n**	Adjusted* **OR (95%CI)**	**p**
FFMQ total	58	0.96 (0.93–1.00)	0.050	51	0.97 (0.91–1.03)	0.384
Non-judging of inner experience	58	0.95 (0.89–1.02)	0.185	51	1.05 (0.92–1.21)	0.446
Describing (items with positive formulation)	59	0.91 (0.81–1.03)	0.147	52	0.86 (0.73–1.02)	0.082
Describing (items with negative formulation)	59	1.00 (0.86–1.17)	0.974	52	1.03 (0.82–1.30)	0.807
Non-reactivity to the inner experience	59	0.91 (0.81–1.01)	0.084	52	0.78 (0.63–0.96)	0.021
Acting with awareness (automatic pilot)	59	0.91 (0.77–1.08)	0.270	52	1.05 (0.82–1.35)	0.667
Acting with awareness (distraction)	59	0.96 (0.82–1.12)	0.601	52	1.15 (0.90–1.47)	0.267
Observing	59	0.94 (0.87–1.02)	0.136	52	0.84 (0.73–0.97)	0.019
Type of hypnotic	62	0.93 (0.34–2.57)	0.894	53	0.62 (0.16–2.47)	0.500
**Lack of compliance**	**n**	**Crude OR (95% CI)**	**p**	**n**	Adjusted* **OR (95%CI)**	**p**
Total FFMQ	69	0.95 (0.92–0.99)	<0.01	61	0.94 (0.89–0.99)	0.025
Non-judging of inner experience	70	0.94 (0.88–1.00)	0.066	61	0.96 (0.86–1.07)	0.464
Describing (items with positive formulation)	71	0.94 (0.84–1.05)	0.275	62	0.93 (0.82–1.05)	0.245
Describing (items with negative formulation)	71	0.92 (0.79–1.07)	0.275	62	0.91 (0.74–1.11)	0.349
Non-reactivity to the inner experience	71	0.93 (0.84–1.02)	0.124	62	0.86 (0.75–0.99)	0.034
Acting with awareness (automatic pilot)	71	0.86 (0.72–1.01)	0.068	62	0.93 (0.75–1.15)	0.494
Acting with awareness (distraction)	70	0.92 (0.79–1.06)	0.238	62	0.94 (0.78–1.13)	0.502
Observing	71	1.04 (0.96–1.12)	0.306	62	1.04 (0.96–1.14)	0.326
Type of hypnotic	74	0.79 (0.31–2.02)	0.629	63	0.67 (0.21–2.12)	0.492

*Models adjusted for insomnia severity, age, schooling, income, and anxiety.

In regression models with “Problematic Use” as an outcome, none of the facets of mindfulness were significantly associated with problematic use of hypnotics, neither in the crude nor in the adjusted models. Regarding “Preoccupation”, often characterized by anticipatory anxiety about non-use of medication, the observing facet and the total score of the FFMQ were significantly and inversely associated with this subscale of the Bendep-SRQ-PV. (Table 4).

In models including only with the women who had attempted to reduce or discontinue hypnotic use, FFMQ subscales “Observing” and “Non-Reactivity to inner experience” were associated with lower scores on the subscale of harm resulting from withdrawal symptoms (Table 4).

Finally, higher scores on the total FFMQ and in the facet “Non-Reactivity to inner experience” reduced the odds of a higher score in the “Lack of compliance” subscale of the Bendep-SRQ-PV (Table 4).

## Discussion

Results from this cross-sectional study revealed two main findings. First, contrary to hypotheses, facets of mindfulness were not significantly associated with the awareness of problematic use of hypnotics. However, aligned with hypotheses, some of the facets were inversely associated with specific aspects of dependence. Specifically, the mindfulness facets “Observing” and “Non-Reactivity to inner experience”, in addition to the total score, were associated with “Preoccupation with the lack of use”, “Lack of compliance with the therapeutic regimen” and “Impairments caused by withdrawal symptoms when discontinuation is attempted”.

To our knowledge, this is the first study to evaluate the relation between dispositional mindfulness and hypnotic dependence. The women interested in participating were characterized by higher educational level and income, representative of the intersection of women who use hypnotics and those who are interested in mindfulness. A recent survey with a representative U.S. sample on use of hypnotics between 2005 and 2010 detected that the increase in the frequency of use of hypnotics was proportional to the increase in level of education [41]. Similarly, the profile of people who searched for mindfulness training and resources in the U.S. was composed of 61% of women who, when compared to non-meditators, were more likely to have at least some college education, and 27% more likely to report having insomnia [42]. It should be noted that, although most of our sample used hypnotics under prescription and reported that their physicians evaluated the impact of the medication on their sleep and/or its deleterious effects on their motor or cognitive functioning, 44.4% reported no evaluation from a professional who renewed their prescription, supporting the chronic and inappropriate use of the medication [43].

Of note, regression models with subscales of dependence as outcomes, adjusted for income, age, schooling, anxiety and insomnia, yielded no significant differences between use of benzodiazepines and non-benzodiazepines (z drugs) on any of the dependence subscales. Although it is not possible to state that dependence is similar between these two classes of hypnotics, our data did not yield a significant difference between the individuals who use these two classes of medication. Previous studies have found evidence that chronic use of z drugs may lead to dose escalation and heavy use, and that the renewal of prescriptions without a proper medical evaluation is associated with a higher risk of dependence [44, 45]. These findings reinforce the importance of including this class of medication in studies that evaluate chronic use of hypnotics.

Regarding our primary analyses assessing the relation between facets of mindfulness and indices of dependence, none of the mindfulness facets showed a significant association with “Problematic Use”. This might indicate that dispositional mindfulness does affect the awareness of risks associated with chronic hypnotics use. These findings may be understood when we consider that the majority of use was under prescription, and prescribed medications are frequently believed to be safe, which may lead users to view use as non-problematic, or as not involving risk [46]. A surprising finding was that the facets “Observing” and “Describing” were not inversely associated with “Problematic Use”. These mindfulness subscales describe the perception and description of present-moment inner experiences, such as sensations, cognitions, and emotions. Users of hypnotics may favor awareness of the acute effect of use on their body over other perceptual experiences [19]. Acknowledging problematic use demands an understanding of the impact of the medication over time, skills that should be developed by other interventions, such as psychoeducation or motivational interviewing, as observed in previous studies with this population [11, 47, 48].

Considering the current study’s focus on the anticipation components of dependence, represented by the subscales “Preoccupation” and “Lack of compliance”, it is notable that the total mindfulness score and the “Observing” facet were the primary protective factors against higher scores on the subscale “Preoccupation”, even after the adjustment of the models. Our findings are in line with previous studies that identified skills of attention and awareness, as measured by the subscale “Observing”, as vital to relapse prevention, when included with the other mindfulness skills of non-judgment, non-reactivity and focused action, comprising the total FFMQ score in populations without experience in meditation [19], as was the case with our sample [17]. Concerning the subscale “Lack of compliance”, the total mindfulness score and the facet “Non-reactivity to inner experience” were the only scores that remained significant after the adjustment of the model as protective factors against higher scores in this subscale. This might suggest the importance of awareness and non-reactivity when facing discomfort, fostering different choices, breaking the vicious circle between trigger and substance use, as observed in previous studies that tested this relation [25].

Finally, the mindfulness facets “Observing” and “Non-Reactivity to inner experience” were the only facets that remained significant after the adjustment as potential protective factors against higher scores on the “Withdrawal” scale. It has been suggested that mechanisms through which mindfulness skills might benefit health are not related to the change in the experience itself, but to the change in one’s relation to experiences, i.e., with a more accepting and open perspective [26]. A recent study demonstrated that “non-reactivity” moderated the effect of “Observing” on the symptoms of anxiety and thought rumination, increasing the use of more adaptive strategies of emotional regulation and reducing maladaptive strategies, such as suppression. Therefore, indirect effects of “Observing” in each symptom grew stronger as “Non-reactivity” increased [35]. Consequently, our results indicate that even though withdrawal symptoms occur upon removal of the hypnotic, women scoring higher on observing and not reacting to symptoms also had a lower score in the subscale of perception of impairments induced by withdrawal.

Broadly speaking, results from the present study corroborate previous findings with different clinical conditions, indicating that dispositional mindfulness is negatively associated with habitual negative thoughts [49] and experiential avoidance [50], in the same way that individuals who present higher levels of mindfulness tend to have the inherent ability to observe their thoughts with detachment, without adhering or reacting to them as if they were an absolute truth, but rather as transient mental events [51].

### Strengths and limitations

Among the strengths of this study, one to be highlighted is the novelty of our findings. Although studies on related topics are common, such as mindfulness and other substances of dependence [22] and mindfulness and insomnia [20], we did not find any studies in the literature that evaluated the relation between dispositional mindfulness and chronic hypnotic use, despite the fact that it is highly prevalent and known to be associated with several issues [3]. Moreover, our findings support the importance and need of further studies on non-pharmacological approaches to the current population, once the lack of this treatments seems to be an important reason to the maintenance of chronic use [8].

Additionally, the instruments used to measure the primary study variables, hypnotic dependence and mindfulness, are multidimensional, promoting a better understanding of which aspects of dependence are potentially mitigated by specific mindfulness skills. The FFMQ is one of the main instruments used worldwide to evaluate mindfulness, having been validated in several countries, including Brazil [34], promoting the replication of the study in different populations.

The primary limitation of the study is its cross-sectional design, preventing inference of causality. Another limitation to consider is bias in the sample. Participation in the study was not through referral from the medical services, widely known for the high number of hypnotic prescriptions, but through media broadcast about a longitudinal clinical trial. Consequently, caution should be used in generalizing results, since the recruitment methods might have attracted individuals who are more aware of their health condition and/or more interested in mindfulness. Another consideration is use of self-report measures, which may be influenced by social desirability, or subject to mistaken interpretations of the content of questions. Finally, the small sample size increases likelihood of Type II error.

Previous studies with similar populations have demonstrated that integrative and group psychotherapeutic strategies, which consider coexisting psychiatric symptoms such as anxiety and insomnia, are useful to individuals in the process of gradual hypnotics tapering [52]. Hence, to further explored results from the current study, and to broaden therapeutic options for this population, further studies with larger samples and/or longitudinal designs evaluating the impact of mindfulness interventions in this population are warranted.

## Conclusion

This is the first study, to our knowledge, to provide data on the relation between dispositional mindfulness and hypnotic dependence among women. Select mindfulness facets were associated with the dimensions of dependence that involve (1) the anticipatory cognitive component related to the possible unavailability of the medication; (2) the compulsive component of using the medication beyond medical prescription, also related to the anxiety of suffering from the symptoms in case the dose indicated does not yield the expected results, and (3) perception of the harm caused by withdrawal symptoms in the event of reduction or withdrawal of the hypnotic. This suggests that dispositional mindfulness was associated with milder levels of dependence and suffering caused by withdrawal symptoms, but not necessarily with the acknowledgment of risks and problematic use. These findings stress the importance of considering positive psychological factors in studies on dependence and chronic use of hypnotics.

## Supporting information

S1 File Database. The database used for the analyses in this study is available in this attachment.
(DTA)
